# Supplementary material for: Effects of Frying Conditions on Volatile Composition and Odor Characteristics of Fried Pepper (Zanthoxylum bungeanum Maxim.) Oil
Source: Foods. 2022 Jun 6;11(11):1661. doi: 10.3390/foods11111661 (PMC9180787; doi:10.3390/foods11111661)
Supplement: Supplementary file 1 [file foods-11-01661-s001.zip › foods-1743558-supplementary.pdf]

Effects of Frying Conditions on Volatile Composition and Odor Characteristics of Fried Pepper (*Zanthoxylum bungeanum Maxim.*) Oil

Ruijia Liu [1], Nan Qi [1], Jie Sun [1,*], Haitao Chen [1], Ning Zhang [1] and Baoguo Sun [1]

Table S1. Mean intensity values of the five attributes for the different frying temperatures.

Sample	herbal	citrus-like	floral	rosin	fatty
FPO1	3.3[a]	3.3[a]	3.0[a]	2.5[a]	2.3[a]
FPO2	3.3[a]	3.3[a]	3.0[a]	1.9[b]	2.6[b]
FPO3	3.3[a]	3.5[b]	3.5[b]	1.4[c]	3.3[c]
FPO4	4.1[b]	2.6[c]	2.0[c]	2.1[d]	4.3[d]

Means within different letters are significantly ($P < 0.05$) different for the same parameter.

Table S2. Mean intensity values of the five attributes for the different frying times.

Sample	herbal	citrus-like	floral	rosin	fatty
FPO5	3.1[a]	3.2[a]	3.2[a]	2.2[a]	3.1[a]
FPO6	3.3[b]	3.5[b]	3.5[b]	1.4[b]	3.3[b]
FPO7	4.0[c]	3.3[a]	2.5[c]	1.4[b]	3.5[c]

Means within different letters are significantly ($P < 0.05$) different for the same parameter.
